# Radiation-induced muscle fibrosis rat model: establishment and valuation

**DOI:** 10.1186/s13014-018-1104-0

**Published:** 2018-08-29

**Authors:** Yue Zhou, Xiaowu Sheng, Feiyan Deng, Hui Wang, Liangfang Shen, Yong Zeng, Qianxi Ni, Shibin Zhan, Xiao Zhou

**Affiliations:** 1 Department of Radiation Oncology, Key Laboratory of Translational Radiation Oncology, Hunan Province. Department of Radiation Oncology, Hunan Cancer Hospital and The Affiliated Cancer Hospital of Xiangya School of Medicine, Central South University, Changsha, Hunan Province China; 2 Department of Head and Neck Surgery, Hunan Cancer Hospital and The Affiliated Cancer Hospital of Xiangya School of Medicine, Changsha, Hunan Province China; 3 Translational Medical Center, Hunan Cancer Hospital and The Affiliated Cancer Hospital of Xiangya School of Medicine, Central South University, Changsha, Hunan Province China; 4 Department of Tumor, Xiangya Hospital of Central South University, Changsha, Hunan Province China; 5 Xiangya School of Medicine, Central South University, Changsha, Hunan Province China; 6 University of South China, Hengyang, Hunan Province China; 7 Central Laboratory, Hunan Cancer Hospital and The Affiliated Cancer Hospital of Xiangya School of Medicine, Central South University, Changsha, Hunan Province China

**Keywords:** Radiotherapy, Radiation damage, Muscle fibrosis, Rat model, Satellite cell

## Abstract

**Background:**

Lack of animal model of radiation induced muscle fibrosis, this study aimed to establish such a model by using 90 Gy single dose irradiation to mimic clinical relevance and also to explore the potential post-irradiation regenerative mechanism.

**Methods:**

SD rats were randomly divided into dose investigation groups and time gradient groups. Group1–6 were irradiated with a single dose of 65Gy, 70Gy, 75Gy, 80Gy, 85Gy and 90Gy respectively, and the degree of rectus femoris fibrosis in the irradiated area was detected at 4 weeks after irradiation. Group 7–9 were irradiated with a single dose of 90Gy, and the results were detected 1, 2, 4, and 8 weeks after irradiation. Then the general condition of rats was recorded. Masson staining was used to detect muscle fibrosis. The ultrastructure of muscles was observed by electron microscope, and the expression changes of satellite cell proliferation and differentiation related genes were detected by quantitative real-time-PCR.

**Results:**

A single dose of 90Gy irradiation could cause muscle fibrosis in rats. As time goes on, the severity of muscle fibrosis and the expression of TGF- β1 increased. Significant swelling of mitochondria, myofilament disarrangement and dissolution, obvious endothelial cell swelling, increased vascular permeability, decrease of blood cell, deposition of fibrosis tissue around the vessel could be found compared with the control group. At around the 4th week, the expressions of Pax7, Myf5, MyoD, MyoG, Mrf4 increased.

**Conclusion:**

Irradiation of 90Gy can successfully establish the rat model of radiation-induced muscle fibrosis. This model demonstrated that regenerative process was initiated by the irradiation only at an early stage, which can serve a suitable model for investigating regenerative therapy for post-radiation muscle fibrosis.

## Background

Radiotherapy is an effective treatment for cancer, and more than 50% of tumor patients need it for radical or palliative treatment [1], for which can be used to treat various tumors, such as nasopharyngeal carcinoma (recommended as the preferred treatment), lung cancer, breast cancer, rectal cancer, etc. Radiation-induced fibrosis (RIF) is a long-term side effect of radiation therapy, and it results in a multitude of symptoms that significantly impact quality of life or even endangers patients’ life [2]. Li Jian observed 267 cases of nasopharyngeal carcinoma after radiotherapy and found that all patients presented neck radiation fibrosis, and about 24.34% patients were of heavy degree. Their post irradiation symptoms included cervical muscle fibrosis, trismus, torticollis, neck muscle weakness, muscle dystonia, shoulder pain and shoulder functional disorder [3]. Many years of our clinical records demonstrate that radiation-induced fibrosis could cause local tissue scar and contracture, leading to difficulty in re-operation for cancer recurrence, while hypoxia in local tissues can result in poor efficacy of re-irradiation. The molecular mechanism of fibrosis induced by RIF is similar to that caused by other damages, such as sports, chemical stimulation, trauma and surgery [4–6]. The main mechanisms are: cellular DNA damage caused by radiation [7, 8], tissue cell or stem cell injury and loss [9], cellular signal pathway alterations such as the activation of TGF-β1 signaling pathway [4, 8], and genetic or epigenetic changes [7, 10, 11]. After injury, muscle tissue undergoes four stages: degeneration, inflammation, muscle regeneration and fibrosis formation. After irradiation, DNA damage, cell apoptosis and cell necrosis occur in tissue cells. And a series of inflammatory mediators are released during inflammatory reactions, including fibrosis promoting factors such as TNFα, IL-1, IL-6 [12], TGF-β1 [13–18], CTGF [19]; and the fibrosis inhibitory factors such as HGF [20], IFN-γ [21] release etc.. This process is also correlated to the activation of skeletal satellite cells(SCs) [22, 23]. SCs are the self-renewal myogenic stem cells in muscles, and can differentiate into new tissue cells.

Normally, SCs are in resting state expressing paired box gene 7 (Pax7). They are activated when the muscle is damaged. Those activated SCs migrate towards the injured site, enter the myogenic differentiation pathway, and express myogenic factor 5 (Myf5) immediately [24]. SCs proliferate furtherly, and some continue to undergo myogenic differentiation and express myogenic determining factor(MyoD). Then the expression of myogenin (MyoG) is followed, which promotes the fusion of myoblasts, and the combination with damaged fibers to repair them [25]. The release of inflammatory cytokines and mediators, and the changes of intracellular signal pathway, induce fibroblast activation and epithelial mesenchymal transition of other precursor cells (vascular endothelial cells) to form myofibroblast [6, 26, 27]. Sustained activation and proliferation of myofibroblasts, promotes collagen secretion and extracellular matrix local deposition [28], eventually leading to the formation of fibrosis [29], and fibrosis also inhibits muscle repair [28].

It can be seen from the above, proliferation and myogenic differentiation of SCs are the key factors for the repair of muscle injury. Therefore, we hypothesize that radiation could damage SCs and cause the difficulty of their activation and proliferation, affect muscle regeneration and repair, and thus change the process of fibrosis formation. And so far, no standardized therapy prevents RIF in muscle, and animal models for therapeutic tests are poorly established [30]. Thus, we establish a rat model of radiation induced muscle fibrosis, and study the proliferation and differentiation of SCs at different stages after irradiation. This provides new ideas and foundation for the treatment of RIF in the future.

## Methods

### Experimental animals

The SD rats used in this experiment were all 6-month-old, weighing about 220–250 g. They were divided into 9 groups (10 mice per group, half male and half female). Researchers involved have received the Central South University animal experiment training and obtained the certificates. The animal tests, housing and environmental conditions were in line with “the Guide for the Care and Use of Laboratory Animals” [31]. Rats were divided into cages (4 rats per cage). To reduce pressure of transportation, checking and adaptation for the new environment, a week of recovery time was given. Meanwhile, their diet, feces, urine and activities were observed every day. The rats were in good health after this adaptation period.

### Establishment of the rat model for radiation-induced muscle fibrosis

Five percent pentobarbital sodium was injected intraperitoneally into the anesthetized rat (0.1 mL/100 g), and the surface of the medial rectus femoris on the left thigh was marked by a marker. And 10 min later, radiation was given by Nucletron Microselectron-HDR Ir-192 afterloading System (Nucletron Company / Nederland), with the applicator tube fixed at the mark on the left thigh. The dose normalization point was 0.5 cm below the source center, and the irradiation area was 0.5 cm radius around the mark point. Group 1 to 6 were respectively given 65Gy, 70Gy, 75Gy, 80Gy, 85Gy, 90Gy single dose of irradiation, and the fibrosis degree on the rectus femoris at the irradiated site was detected 4 weeks after irradiation. Group 7 to 9 were given 90Gy single dose of irradiation, and the results were detected at the 1st, 2nd, 4th and 8th week after irradiation respectively.

### General conditions

The diet, feces, urine, activity and skin condition of rats were observed, and the number of dead rats was recorded.

### Masson staining

The irradiated rectus femoris muscle was fixed by 4% formaldehyde for 24 h, and then slices of paraffin-embedded biopsy were produced. Masson trichrome staining (Baso, BA-40798) was used after slice dewaxing. The inverted microscope (Zeiss, Axio Scope A1) was used for observation. All visual fields in each slice were selected, and the percentage of collagen fibrosis area was analyzed by Image-Pro Plus 6 software (Media Cybernetics, Bethesda, USA).

### Electron microscopy

The rectus femoris at irradiated site was embedded in chenodeoxycholic acid, and 24 h later tissue sections were carried out. The ultrastructure of vascular endothelial cells, muscle fibers and mitochondria were observed by Tecnai G2 Spirit and electron microscopy (FEI, Oregon, Hillsboro, USA). GATAN ORIUS CCD CAMERA (GATAN, Coronado Lane, Pleasanton, CA, USA) was used to collect images.

### Real time RT-PCR

The primers were designed by NCBI Primer-Blast and synthesized by Shanghai biological engineering company. The rectus femoris of group 7–9 were irradiated, then the RNA was extracted. First-strand cDNA was synthesized from 1 mg total RNA with a First Strand cDNA Synthesis Kit (Thermo Scientific, Massachusetts, USA). Total cDNA was amplified for over 45 cycles in a system, which contained SYBR Green I TaqMan probes (Roche, Basel, Switzerland). Relative gene expression was calculated with 2^–△△CT^ method, and GAPDH was used as housekeeping gene reference for normalization. The expression of PAX7, MyoD, MyoG, Mrf4, Myf5 were detected. The primer sequence was as follows (Table 1).

**Table 1:** Primer sequence of the marker genes

Gene name	Upstream primers	Downstream primers
GAPDH	AGGTCGGTGTGAACGGATTTG	TGTAGACCATGTAGTTGAGGTCA
PAX7	GAGTATAAGAGGGAGAACCCCG	TTGATTCTGAGCACTCGGCTAA
MyoD	GCTCTGATGGCATGATGGATTAC	CTATGCTGGACAGGCAGTCG
MyoG	ACTACCTTCCTGTCCACCTTCA	AGGCCTCATTCACTTTCTTGAG
Mrf4	ACAGCTACAAACCCAAGCAAGA	CTTGCTCCTCCTTCCTTAGCAG
Myf5	TCTGATGGCATGCCTGAATGTAA	AAGGAGCTCTTATCTGAAGCACA

### Western blot

The total protein was extracted from the cell lysate of the rats’ rectus femoris. 10%SDS polyacrylamide gel was configured and 20 μg protein was added before electrophoresis. After electrophoresis, the protein was transferred to the PVDF membrane. 5% of the skimmed milk was closed for 1 h, and the first antibody (TGF-β1, GAPDH, 1:1000 dilution) was incubated overnight at 4 °C, then the membrane was washed. Then it incubated with the second antibody (The sheep anti-rabbit IgG conjugated to horseradish peroxidase; 1: 3000 dilution) at room temperature for 1 h, and the enhanced chemiluminescence(ECL) was performed after the membrane was washed.

### Statistical analysis

All data are represented by mean ± standard error (mean ± SEM). The experimental data was processed by SPSS 13.0 software (SPSS, Chicago, IL, USA). The difference between groups was analyzed by t-test, and *p* < 0.05 means statistically significant.

## Results

### Masson staining of each dose group

To determine the appropriate radiation dose for the establishment of the rat model for radiation-induced muscle fibrosis, the Ir-192 high-dose-rate afterloading unit was used. Single dose was performed on the thigh rectus femoris of SD adult rats in the following dose groups: group 65Gy, group 70Gy, group 75Gy, group 80Gy, group 85Gy, group 90Gy. Muscle fibrosis was characterized by the increased secretion of collagen fibers and the deposition of extracellular matrix [28]. TGF- β1 is an important fibrosis promoting factor [13–18]. Muscle fibrosis usually occurs 2–4 weeks after injury, and becomes more serious as time goes on [28]. To confirm whether the muscle fibrosis model was formed after radiation exposure and to identify the effect of the model, masson staining was used to detect the fibrosis degree at the irradiated site 4 weeks after radiation.

**Fig. 1:**
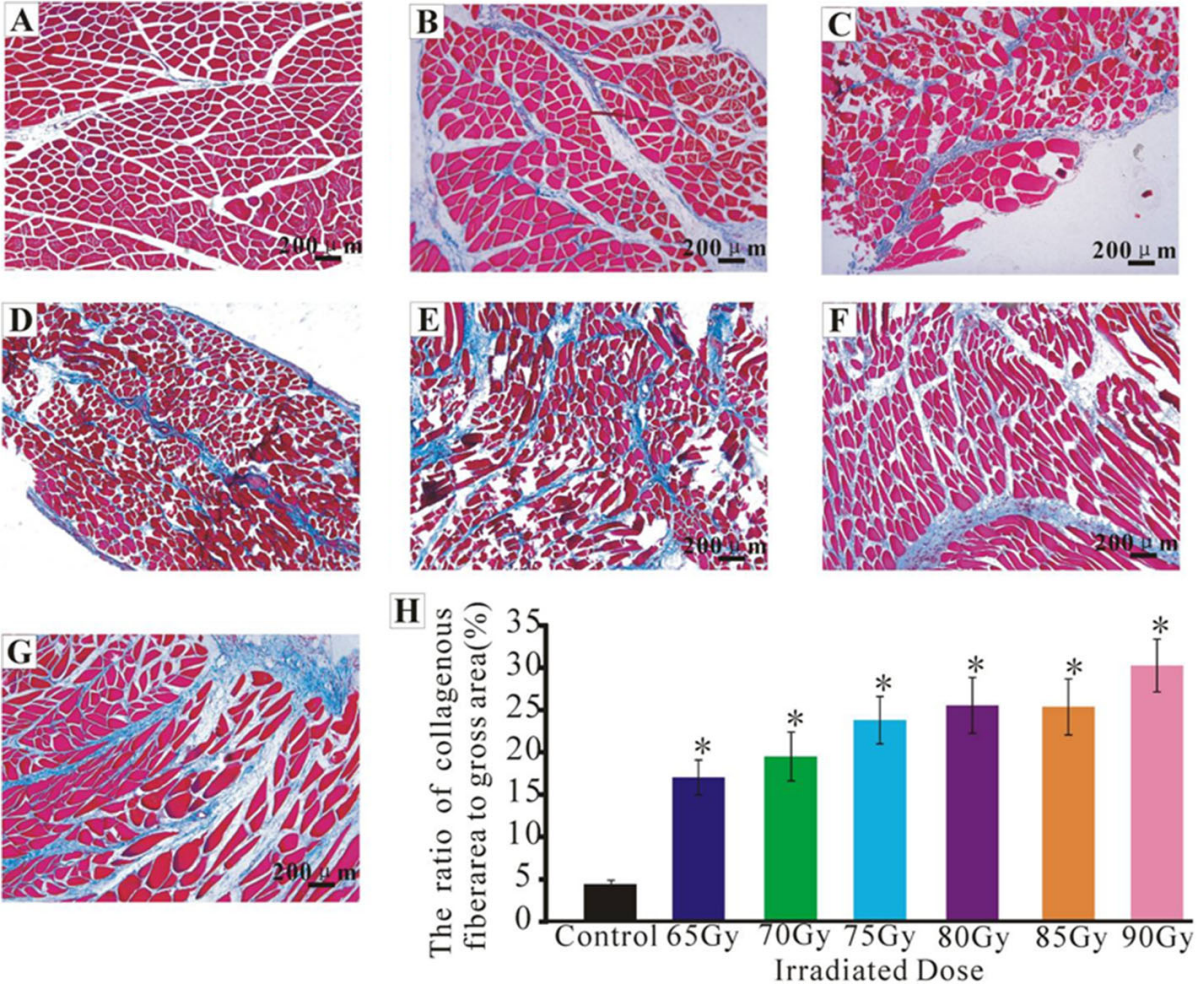
Four weeks after irradiation, the Masson staining of rectus femoris muscle in each dose group. The collagen fibers were blue and the muscle fibers were red. **a** Control; **b** 65Gy; **c** 70Gy; **d** 75Gy; **e** 80Gy; **f** 85Gy; **g** 90Gy; **h**. analysis of collagen fiber area percentage in each dose group, *p < 0.05, compared with the control group. The area and percentage of muscular fibrosis increased with dose

The rats were divided into 6 dose groups: 65Gy, 70Gy, 75Gy, 80Gy, 85Gy, 90Gy. Four weeks after single irradiation of each dose on left rectus femoris, the muscle tissue biopsies were made and stained with Masson. Collagen fibers were blue, and muscle fibers were red. The percentage of fibrosis area increased with increasing dose. In control group, the percentage of fibrosis area was 4.43±0.46 (*n* = 10, Fig. 1a). In 65Gy group, it was 17.03±2.06(*n* = 10, Fig. 1b). In 70Gy group, it was 19.48±2.88(*n* = 10, Fig. 1c). In 75Gy group, it was 23.85±2.80(*n* = 10, Fig. 1d). In 80Gy group, it was 25.45±3.29(*n* = 10, Fig. 1e). In 85Gy group, it was 25.31±3.30(*n* = 10, Fig. 1f). In 90Gy group, it was 30.15±3.13(*n* = 10, Fig. 1g). The fibrosis area of 65Gy–90Gy group was higher than that of control group (*p* < 0.001, Fig. 1h).

### General performance of rats after 90Gy single dose irradiation

To achieve stable effect, a single dose of 90Gy was selected for the model according to the Masson staining results. After irradiation with 90Gy radiation on the rectus femoris of SD adult rats, intermittent claudication occurred in very few rats (non-statistical data). No death was found, the feces and urine were normal, and no significant weight loss or hair loss occurred.

### Muscle fibrosis increased with time after 90Gy single dose irradiation

**Fig. 2:**
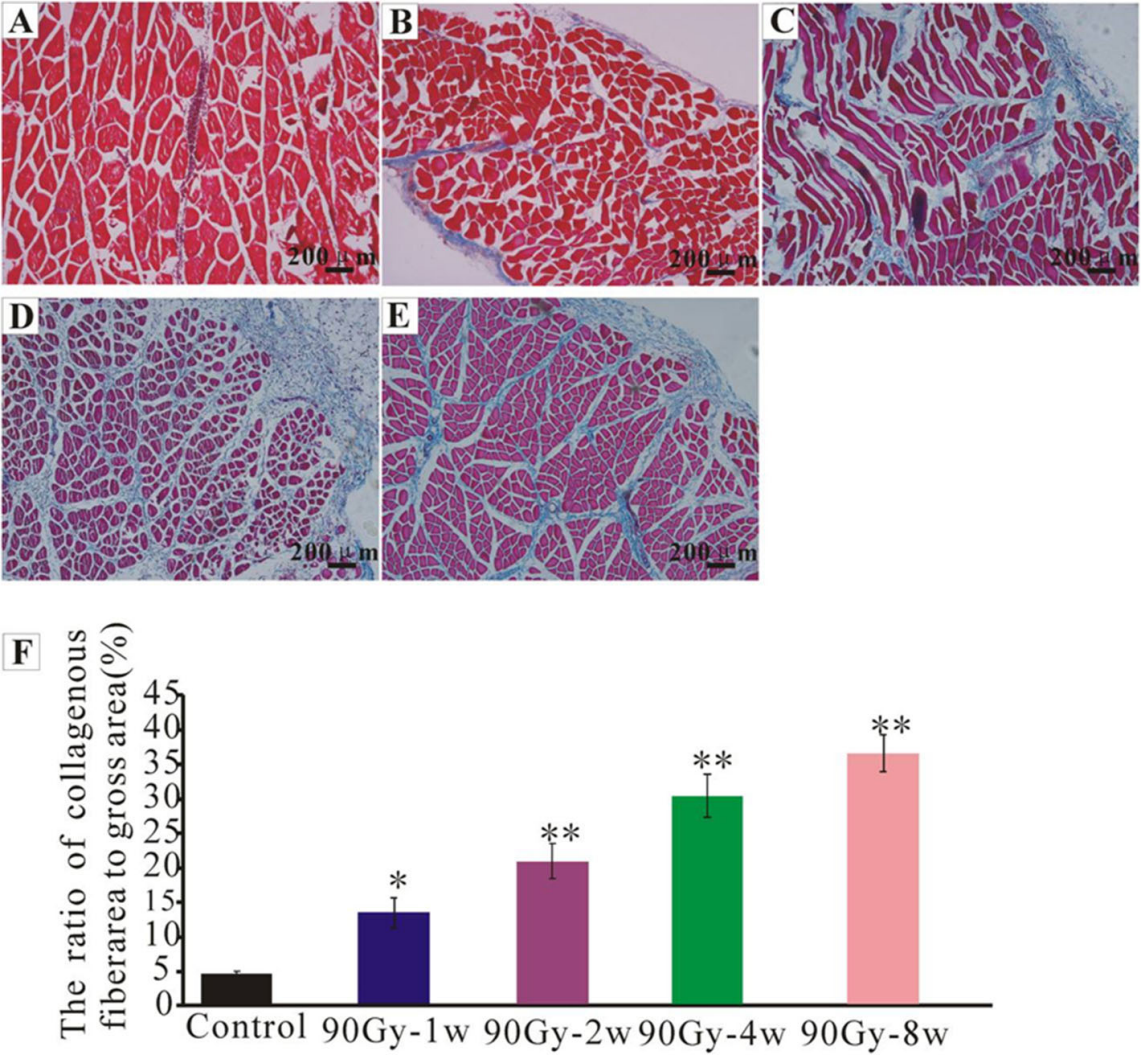
Masson staining at different time points after 90Gy irradiation. The collagen fibers were blue and the muscle fibers were red. **a** control group; **b** 90Gy-1w group; **c** 90Gy-2w group; **d** 90Gy-4w group; **e** 90Gy-8w group; **f**. analysis of the collagen fiber area percentage in each group, *p < 0.05, **p < 0.01, compared with the control group. Muscle fibrosis aggravated with time

One week, 2 weeks, 4 weeks and 8 weeks after 90Gy irradiation, the rectus femoris paraffin sections of the irradiated area were taken and stained with Masson. Collagen fibers were almost invisible in the control group (no radiation exposure) (4.43±0.46, *n* = 10, Fig. 2a). Fibrosis area percentage in 90Gy-1w group increased comparing with the control group, but the amount was less (13.25±2.22, *n* = 10, Fig. 2b). Fibrosis area percentage in 90Gy-2w group increased comparing with the control group (20.73±2.53, *n* = 10, Fig. 2c). Fibrosis area percentage in 90Gy-4w group increased comparing with the control group (30.15±3.13, *n* = 10, Fig. 2d). Fibrosis area percentage in 90Gy-8w group increased comparing with the control group (36.36±2.73, *n* = 10, Fig. 2e).

### TGF-β1 increased with time in the process of irradiation induced fibrosis

**Fig. 3:**
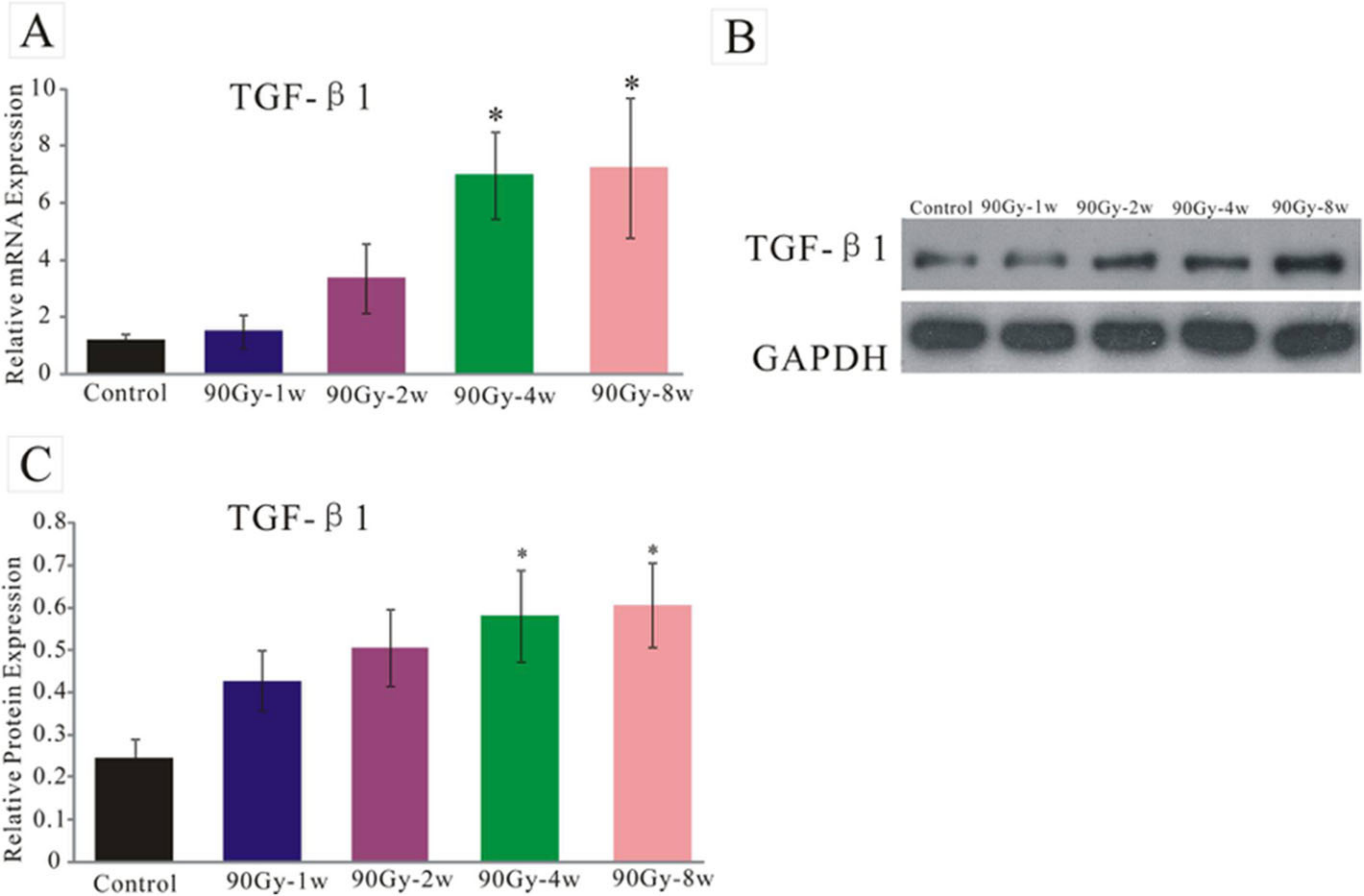
**a** TGF-β1 mRNA expression level on 1 week, 2 weeks, 4 weeks and 8 weeks after 90Gy radiation(*p < 0.05). **b** Expression level of TGF-β1 protein on 1 week, 2 weeks, 4 weeks and 8 weeks after 90Gy radiation. **c** Expression level of TGF-β1 protein on 1 week, 2 weeks, 4 weeks and 8 weeks after 90Gy radiation(*p < 0.05). The expression of TGF- β 1 increased with time

As an important fibrosis promoting factor, TGF-β1 mRNA expression level increased during the pathological process of fibrosis. The mRNA expression level of TGF-β1 was measured 1 week, 2 weeks and 4 weeks after 90Gy irradiation. No significant differences of TGF-β1 mRNA expression were found between 90Gy-1w group and the control group (1.53±0.58, *n* = 6, *p* > 0.05). Comparing with the control group, there was a growing trend of TGF-β1 mRNA expression in 90Gy-2w group, but with no statistical significance (3.34±1.22, *n* = 6, *p* > 0.05). TGF-β1 mRNA expression in 90Gy-4w group was more than that of control group (6.97±1.53, *n* = 9, *p* < 0.05) (Fig. 3a). TGF-β1 mRNA expression in 90Gy-8w group was more than that of control group (7.25±2.45, *n* = 9, *p* < 0.05) (Fig. 3a). Comparing with the control group there was an increasing tendency of TGF-β1 protein expression (Fig. 3b and c).

### Ultrastructure of muscle tissue

**Fig. 4:**
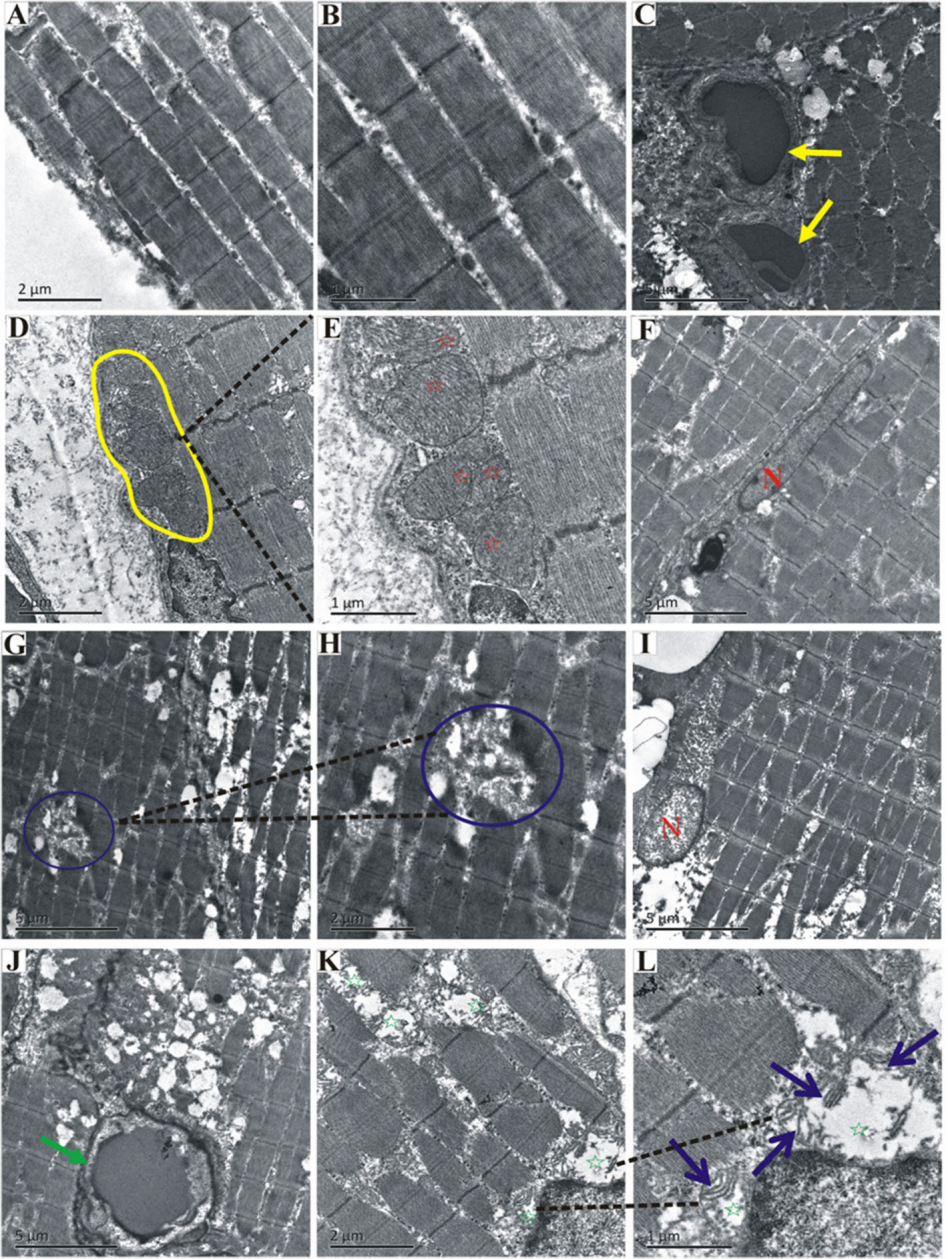
Ultrastructure of muscle tissue detected by electron microscopy. **a**-**f**. Control group. **a**-**b**. Myofilaments. **c** Vascular endothelium. **d**-**e**. Mitochondrion. **f** Nuclei of myofilament. **g**-**l** 90Gy-4w group. **g**-**h** myofilament dissolving. **i** Swollen nuclei of myofilament. **j** Vascular endothelium. **l** Mitochondrial vacuolization

Four weeks after the 90Gy radiation, the rectus femoris tissue of irradiated area was examined by electron microscopy. In the control group, the muscle filaments were arranged neatly (Fig. 4a and b), and the vascular endothelial shape was regular (Fig. 4c yellow arrows). Clear and obvious undamaged mitochondrial crista structures could be seen (Fig. 4d yellow circle, and Fig. 4e red stars). The shape of skeletal muscle nuclei was regular (Fig. 4f letter “N”). In 90Gy-4w group, the arrangement of muscle filaments was disordered, and the filament dissolved (Fig. 4g and h). Swollen skeletal muscle nuclei could be found (Fig. 4f letter “N”). The vascular endothelium swelled and was irregularly shaped (Fig. 4j green arrow). The linear structures were mitochondrial crista (Fig. 4l blue arrows), and the white area around (Fig. 4k and l green stars) was due to loss of it. Vacuolization of damaged mitochondria could be seen (Fig. 4k and l green stars).

### Vascular injury

**Fig. 5:**
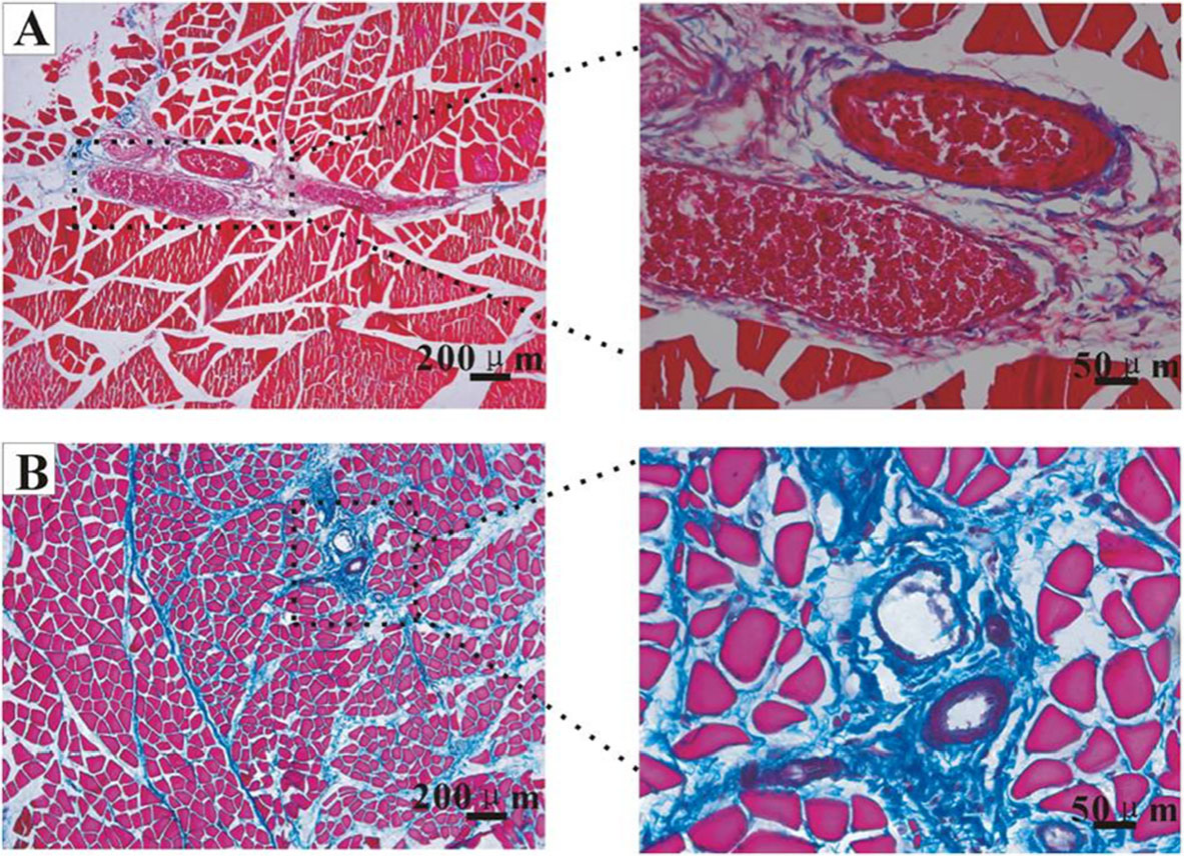
Four weeks after 90 Gy irradiation, Masson staining was used to detect vascular injury. **a** Normal group. The vessel had a regular shape with lots of blood cells in the lumen; **b** 90Gy-4w group. In the arterial lumen, blood cells reduced, and tube wall was thickened. In the venous lumen, blood cells disappeared. A large amount of collagen fibers proliferated around the vessels

Four weeks after 90Gy radiation, Masson staining was performed on the rectus femoris tissue at the irradiated site. In the normal group, many blood cells could be seen in the lumen of the vessel (Fig. 5 A partly enlarged sectional view). In 90Gy-4w group, the blood cells decreased and even disappeared from the vessel lumen, with collagen fiber hyperplasia around the blood vessels (Fig. 5b partly enlarged sectional view).

### Gene expression involved in muscle regeneration

Skeletal SCs are the myoblast stem cells in the muscle, playing an important role in the repair of muscle damage [32].

The genetic expression related to skeletal muscle satellite cells was detected at different time points after 90Gy radiation.

Pax7: 1 week after irradiation, Pax7 mRNA expression was higher than the control group, with no statistical significance (2.14±0.57, *n* = 7, *p* > 0.05). Two weeks after irradiation, its expression was higher than the control group, with no statistical significance (2.39±0.74, *n* = 5, *p* > 0.05). Four weeks after irradiation, its expression was higher than the control group, with statistically significance (3.96±0.90, *n* = 10, *p* < 0.05). Eight weeks after irradiation, there was no difference in its expression compared with the control group (0.67±0.24, *n* = 8, *p* > 0.05);

Mrf5: 1 week after irradiation, Myf5 mRNA expression was lower than the control group, with statistical significance (0.42±0.10, *n* = 8, *p* < 0.05). Two weeks after irradiation, its expression was lower than the control group, with statistical significance (2.82±0.64, *n* = 6, *p* < 0.05). Four weeks after irradiation, its expression was higher than the control group, with statistical significance (3.61±0.87, *n* = 8, *p* < 0.05). Eight weeks after irradiation, there was no difference in its expression compared with the control group (1.38±0.34, *n* = 8, *p* > 0.05);

MyoD: 1 week after irradiation, there was a decreasing trend of MyoD mRNA expression comparing with the control group, with no statistical significance (0.74±0.25, *n* = 7, *p* > 0.05). Two weeks after irradiation, its expression was lower than the control group, with statistical significance (2.45±0.30, *n* = 6, *p* < 0.05). Four weeks after irradiation, its expression was higher than the control group, with statistical significance (5.06±1.30, *n* = 11, *p* < 0.05). Eight weeks after irradiation, there was no difference in its expression compared with the control group (0.61±0.17, *n* = 8, *p* > 0.05);

MyoG: 1 week after irradiation, MyoG mRNA expression was lower than the control group, with statistical significance (0.45±0.10, *n* = 7, *p* < 0.05). Two weeks after irradiation, its expression was not different from the control group (1.49±0.32, *n* = 6, *p* > 0.05). Four weeks after irradiation, there was an increasing trend in the expression compared with the control group, with no statistical difference (2.91±1.14, *n* = 7, *p* > 0.05). Eight weeks after irradiation, there was no difference in its expression compared with the control group (1.24±0.33, *n* = 8, *p* > 0.05);

Mrf4: 1 week after irradiation, Mrf4 mRNA expression was lower than the control group, with statistical significance (0.55±0.12, *n* = 7, *p* < 0.05). Two weeks after irradiation, Mrf4 mRNA expression was lower than the control group, with statistical significance (2.82±0.56, *n* = 6, *p* < 0.05). Four weeks after irradiation, Mrf4 mRNA expression was higher than the control group, with statistical significance (5.73±1.39, *n* = 9, *p* < 0.05). Eight weeks after irradiation, Mrf4 mRNA expression was higher than the control group, with statistical significance (2.48±0.53, *n* = 8, *p* < 0.05).

## Discussion

Commonly seen months or years after radiotherapy of head and neck tumors, radiation-induced fibrosis (RIF), a late complication of radiotherapy, is irreversible. Clinically, RIF is usually caused by high doses of fractionated radiotherapy, and its symptoms include skin ulcers, scarring, muscle atrophy and limited joint activity. Chen et al. [33] found radiation induced trismus for nasopharyngeal carcinoma patients progressed over time. Safora Johansen [34] reported that radiotherapy for breast cancer patients may lead to arm/shoulder pain, restricted arm/shoulder mobility, fibrosis and breast cancer-related lymphedema. However, some previous studies showed that single high dose could induce similar effect on animal models. Gallet P reported 30Gy cobalt 60 irradiation could cause radiation-induced skin and muscle fibrosis in rats [35]. Xinchu Ni reported that a single dose of radiation (80Gy) from a linear accelerator was sufficient in establishing a rabbit model of skeletal muscle injury [36]. HSU et al. indicated that the Wistar rats, when exposed to 80Gy X-rays from a linear accelerator, displayed muscle cell injury [37]. But these experiments of animal models rarely described the RIF symptoms, and the reported doses deviated from clinical ones. Moreover, different radiotherapy equipments also resulted in variations of fibrosis forming efficiency. In addition, radiation-induced fibrosis is characterized by fibroblasts proliferation, myofibroblast differentiation, and synthesis of collagen, proteoglycans and extracellular matrix [29, 38]. In previous studies, those reported animal models could not reach a similar level. In clinics, radiation induced tissue fibrosis process includes skin induration and thickening, muscle shortening and atrophy, and so on [39]. To build an effective and clinical relevant animal model, we explored different doses and found that 90Gy was appropriate, because this dose caused typical pathological findings in accordance with human’s, including inflammation, fibroblast recruitment and extracellular matrix deposition [2]. In addition to muscle fibrosis, we also observed that that a few days after the radiotherapy, severe skin ulcers occurred in rats. Then skin scarring appeared, and intermittent claudication happened, conforming to clinical manifestation. Therefore, 90Gy irradiation was chosen to construct the skeletal fibrosis model. Although irradiation as low as 65Gy could induce skeletal muscle fibrosis, the dosage below 90Gy failed to represent clinical relevant pathological process.

In a previous study, Wei Sun [40], reported that collagen fibers gradually increased in skeletal muscles with the time extension after irradiation. Fibrosis occurred 2–3 weeks after injury, and its area enlarged over time [28]. Our data showed that fibrosis began to appear 1 week after 90Gy irradiation, and its percentage increased over time. TGF-β1 has been considered a key profibrotic cytokine. As two features of radiation-induced fibrosis, DNA damage and inflammation lead to the activation of TGF-β1 and canonical WNT/β-catenin pathway [41]. TGF-β1 is essential for the differentiation of fibroblasts into myofibroblasts [42]. As non-muscle cells, myofibroblasts can contract and relax, and induce an active retraction of granulation tissue, leading to fibrosis [43–45]. Critical for cellular fibrosis in wound as well as numerous organs like kidney, heart, lung, and liver, myofibroblasts play an important role in the phenomenon of contraction- retraction that lasts without relaxation, with an irreversible retraction favored by the synthesis of collagen [41]. Besides, in injured skeletal muscle, TGF-β1 can also induce differentiation of myoblasts into fibrotic cells [46]. TGF-β1 expression gradually increases in a dose-dependent manner and peaks 4 weeks after irradiation [47]. In our study, fibrosis percentage ((Fig. 2) and TGF-β1 expression (Fig. 3) increased with time after irradiation. It is known that TGF-β1 promotes myofibroblast differentiation contributing to RIF, so TGF-β1 may to be proposed as a major target for antifibrotic agents [41, 48, 49].

In addition to TGF- β1, energy metabolism system may also be affected by radiation damage, as it plays an important role in the contraction and relaxation of skeletal muscle [50]. The mitochondria of skeletal muscle are closely related to energy metabolism and muscular function, as they are both the sites of oxidative phosphorylation to synthesis ATP providing energy, and the important organelles regulating intracellular calcium concentration [51]. Recent studies suggest mitochondria may be susceptible to radiation, and could be a source of radiation-induced oxidative stress [52, 53]. The results of the ultrastructure of the experiment (Fig. 4) showed that, there were vacuolated changes of mitochondria in the skeletal muscle cells after irradiation, indicating cell damage and the energy metabolism disorder caused by mitochondria damage. Damage to blood vessels and subsequent hypoxia and ischemia are known to contribute to severe tissue injury such as fibrosis and necrosis [54]. Vascular fibrosis after radiotherapy contributes to severe normal tissue damage and, in some cases, may be a vital prognosis in patients [55]. Irradiation could cause vasculature injury, which is a clinical problem, and endothelial cell damage, which is characterized by increased vascular permeability and endothelial cell apoptosis [56–58]. A few hours and weeks after irradiation, it was observed that endothelial cells would swell with edema, lymphocyte adhesion and infiltration, and endothelial cell apoptosis, which caused vasculature barrier function to lose, integrity to change and permeability to increase [58]. In our study, 4 weeks after irradiation we found endothelium cells swelling (Fig. 4) and vascular permeability changes induced by radiation, with fibrosis formation and fibrins deposition around the vasculature (Fig. 5) in this process. These phenomena indicated that vasculature injury may play a role in muscular fibrosis formation.

**Fig. 6:**
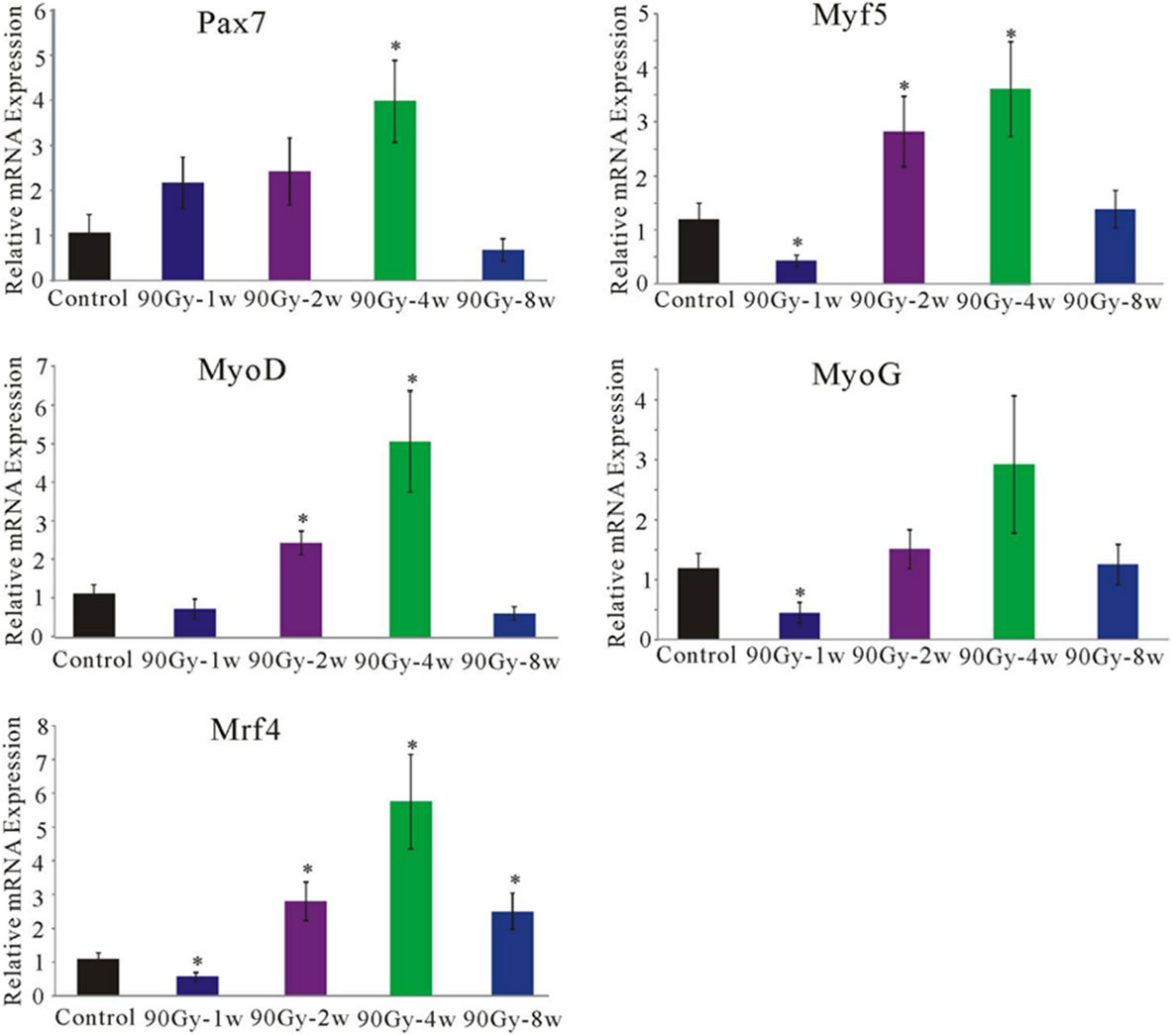
At different time points after 90Gy irradiation, gene expressions (Pax7, Myf5, MyoD, MyoG and Mrf4) related to skeletal muscle satellite cell proliferation and myogenic differentiation in the rectus femoris. (*p < 0.05 compared with the control group.) The expression of Pax7 increased in first week, peaked the fourth week, and fell to a normal level at the eighth week. Mrf5, MyoD, MyoG dropped in the first week, then increased peaking on the fourth week, and fell to a normal level at the eighth week. Mrf4 dropped in the first week, then increased peaking on fourth week, and fell to a level higher than normal at the eighth week

Enhancing muscle regeneration maybe a good strategy to overcome the fibrosis caused by radiotherapy. For this reason, we have carried out a detailed study on the genes related to muscle regeneration. Muscle satellite cells are an important cell source for muscle regeneration, and they differentiate into muscle cells through upregulating Pax7 and myogenic regulatory factors (MRFs). Quiescent Skeletal muscle satellite cells are in G0 phase, implying their resistance to radiation [59]. Pax7 is specifically expressed in proliferating myoblasts, controlling the activation and migration of satellite cell precursors [60]. Pax7 act as master regulators of early lineage specification and are expressed genetically upstream of MRFs during early muscle development [61]. And the expression of MRFs consisting of MyoD, Myf5, MyoG and Mrf4 characterizes various phases of skeletal muscle development including myoblast proliferation, cell-cycle exit, cell fusion and the maturation of myotubes to form myofibers [62]. Our data (Fig. 6) showed that 1 week after 90Gy irradiation, the expression of Pax7 were up regulated, indicating satellite cell enrichment. And the decrease in expression of MRFs may due to the radiation damage to myoblasts, causing myoblasts to decrease. At the 2nd and 4th week after irradiation, myogenic gene expressions were upregulated, indicating muscle regeneration enhancement. It is known that the Pax7 expressed in satellite cells are downregulated during muscle cell differentiation [63]. The primary MRFs including Myf5 and MyoD commit cells to a myogenic program, and the secondary MRFs consisting of MyoG and Mrf4 are terminal differentiation genes that are important for fusion of myocytes and formation of myotubes [64]. In our study (Fig. 6), 8 weeks after radiotherapy, Pax7 and MRFs except for Mrf4 fell to a state similar to that of normal muscles. And this may be that some myoblasts continue to differentiate, fuse and mature. A part of myoblast or activated skeletal muscle satellite cells return to resting state, then muscle regeneration stopped. This data indicated that satellite cells can be activated and differentiate for muscle regeneration after irradiation. But muscle regeneration after radiation was not enough to counteract the fibrosis caused by it. It showed that 8 weeks of 90gy can effectively organize the intrinsic regenerative ability of the muscles, so the model is established successfully. In the future, muscle regeneration can be explored from this model, such as local or systemic injection of stem cells, growth factors, and so on. We also suggest that these actions should be taken immediately after irradiation. This may be an effective prevention for radiation-induced fibrosis, by enhancing muscle regeneration, and inducing powerful satellite cell activation and differentiation.

## Conclusions

This study established a rat model of irradiation induced muscle fibrosis with 90Gy dosage. The severity of muscle fibrosis and the expression of TGF-β1 increased with the time after radiation was demonstrated. And it was found that radiation damaged vascular endothelial cells, and deposition of fibrosis tissue around the vessel was found. Therefore, vascular endothelial injury may play an important role in the formation of fibrosis. Activation and myogenic differentiation of skeletal muscle satellite cells occurred after radiation, leading to muscle regeneration, though not enough to counterbalance the formation of fibrosis. In the future, we would research the relationship between vascular endothelial injury and the formation of radiation-induced muscle fibrosis, and look for a way to promote the activation, proliferation, migration and differentiation of skeletal muscle satellite cells, enhancing muscle regeneration.

## Abbreviations

CSs: Satellite cells
CTGF: Connective tissue growth factor
GAPDH: Glyceraldehyde 3-phosphate dehydrogenase
HGF: Hepatocyte growth factor
IFN-γ: Interferon γ
IL-1: Interleukin 1
IL-6: Interleukin 6
Mrf5: Myogenic factor 5
MRFs: Myogenic regulatory factors
MyoD: Myogenic determining factor
MyoG: Myogenin
Pax-7: Paired box protein 7
PVDF: Polyvinylidene difluoride
RIF: Radiation-induced fibrosis
SDS: Sodium dodecyl sulfate
TGF-β1: Transforming growth factor beta 1
